# Pathogenetic therapeutic approaches for endocrine diseases based on antisense oligonucleotides and RNA-interference

**DOI:** 10.3389/fendo.2025.1525373

**Published:** 2025-01-29

**Authors:** Olga Golounina, Ildar Minniakhmetov, Ramil Salakhov, Rita Khusainova, Ekaterina Zakharova, Igor Bychkov, Natalia Mokrysheva

**Affiliations:** ^1^ Department of Clinical Endocrinology, Endocrinology Research Centre, Moscow, Russia; ^2^ Laboratory of Genomic Medicine, Endocrinology Research Centre, Moscow, Russia; ^3^ Selective Screening Laboratory, Research Centre for Medical Genetics, Moscow, Russia; ^4^ Laboratory of Experimental Gene Therapy for Inherited Metabolic Diseases, Research Centre for Medical Genetics, Moscow, Russia

**Keywords:** endocrine diseases, molecular therapy, RNA therapeutics, antisense oligonucleotides (ASOs), small interfering RNA (siRNA), N-of-1, personalized medicine

## Abstract

Molecular therapy uses nucleic acid-based therapeutics agents and becomes a promising alternative for disease conditions unresponsive to traditional pharmaceutical approaches. Antisense oligonucleotides (ASOs) and small interfering RNAs (siRNAs) are two well-known strategies used to modulate gene expression. RNA-targeted therapy can precisely modulate the function of target RNA with minimal off-target effects and can be rationally designed based on sequence data. ASOs and siRNA-based drugs have unique capabilities for using in target groups of patients or can be tailored as patient-customized N-of-1 therapeutic approach. Antisense therapy can be utilized not only for the treatment of monogenic diseases but also holds significant promise for addressing polygenic and complex diseases by targeting key genes and molecular pathways involved in disease pathogenesis. In the context of endocrine disorders, molecular therapy is particularly effective in modulating pathogenic mechanisms such as defective insulin signaling, beta-cell dysfunction and hormonal imbalances. Furthermore, siRNA and ASOs have the ability to downregulate overactive signaling pathways that contribute to complex, non-monogenic endocrine disorders, thereby addressing these conditions at their molecular origin. ASOs are also being studied worldwide as unique candidates for developing therapies for N-of-1 therapies. The sequence-specific ASOs binding provides exceptional accuracy in N-of-1 approaches, when the oligonucleotide can be targeted to a patient’s exact mutant sequence. In this review we focus on diseases of the endocrine system and discuss potential RNA-targeted therapeutic opportunities in diabetes mellitus, including monogenic beta cell diabetes, and obesity, including syndrome obesity and monogenic obesity, as well as in non-monogenic or complex endocrine disorders. We also provide an overview of currently developed and available antisense molecules, and describe potentials of antisense-based therapeutics for the treatment of rare and «ultrarare» endocrine diseases.

## Introduction

Molecular therapy uses therapeutic agents based on nucleic acids to combat human diseases. This therapy includes strategies such as replacing defective genes, using small interfering ribonucleic acid (siRNA), microRNA (miRNA), or antisense oligonucleotides (ASOs) silencing harmful mutated genes within cells or overexpressing genes through gene supplementation and editing the patient’s genome. Molecular therapy has become a promising alternative for disease conditions unresponsive to traditional pharmaceutical approaches (1). Initially, molecular therapy focused on genetic disorders, especially monogenic diseases, which are classified as orphan diseases because of their rarity (2). This has led to major advances in the field and the availability of molecular therapy products.

Further studies of the etiology of polygenic diseases have revealed new therapeutic targets and offered new opportunities for their treatment. A better understanding of molecular mechanisms has laid the foundation for personalized molecular therapy (3).

In addition, gene expression can be regulated post-transcriptionally using synthetic nucleic acid molecules, preventing splicing, translation or RNA degradation without altering the genetic material of the cell. This approach is particularly effective in diseases caused by loss-of-function mutations (4).

Currently, there are more approved RNA therapies than gene complement therapies. We are entering a new era of innovative RNA therapy using ASOs, siRNAs and miRNAs. These oligonucleotide technologies are successfully used as molecular research tools and therapeutic agents in cellular, preclinical and clinical studies (5–7).

The concept of designing oligonucleotides for binding to specific sequences in target RNA through Watson-Crick base pairing, along with the term «antisense», was first introduced in 1978 by Zamecnik PC. and Stephenson ML. A 13-deoxynucleotide ASO sequence complementary to Rous sarcoma virus RNA has been shown to effectively inhibit viral RNA translation and virus production (8, 9). A year later, a significant post-binding pathway involving the degradation of RNA by ribonuclease H (RNase H) was established (10). In addition, modification of pre-mRNA splicing by steric block, another major mechanism of ASOs action, was reported in 1993 (11).

ASOs are single stranded short nucleic acid oligomers, typically 18–30 nucleotides, that are used to modulate gene expression primarily by specifically binding to complementary sequences of their target RNAs (12). The specificity of this binding is provided by Watson-Crick base pairing, while the entire ASO sequence serves to identify the target RNA. However, theoretically, only 13–15 adjacent nucleotides are sufficient to ensure specificity for a single RNA molecule (13).

**Figure 1:**
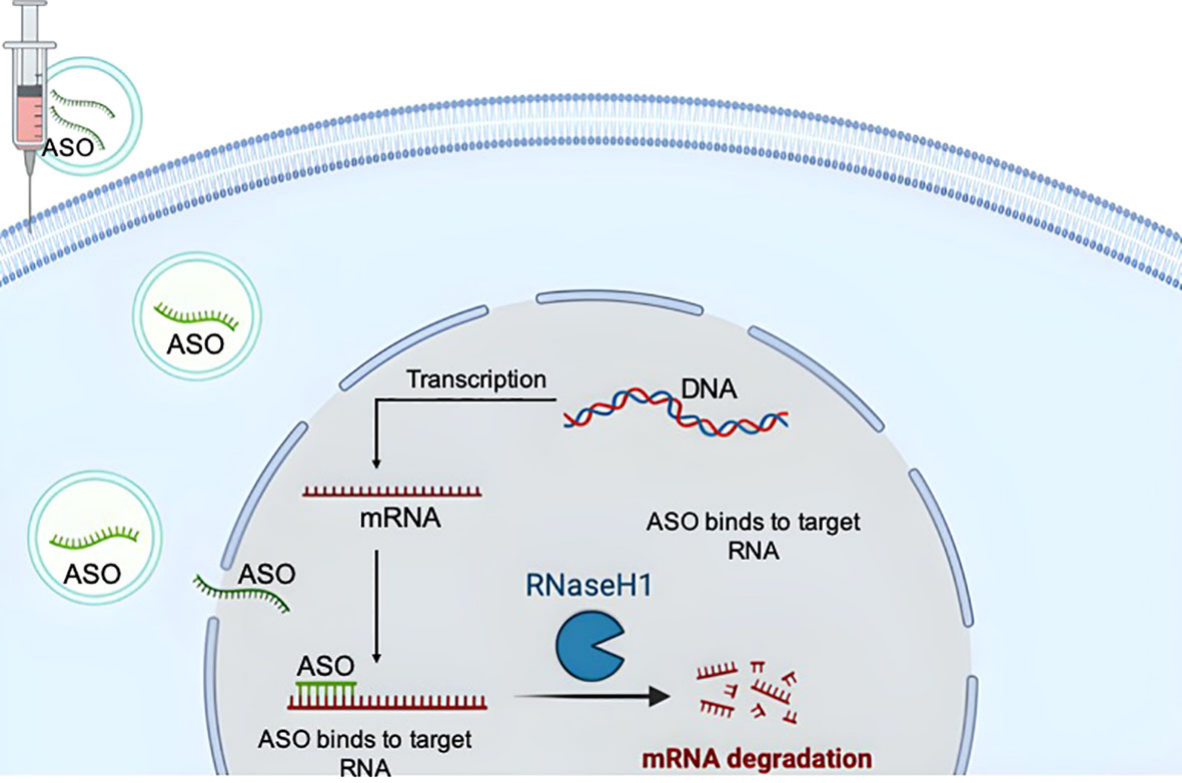
Basic principles of the action mechanisms of antisense oligonucleotides (ASOs). The drawing was created using BioRender web-tool.

Antisense therapy uses synthetic RNA-like oligonucleotides to treat diseases by modulating protein expression of specific target genes. These ASOs are synthetic compounds designed to reproduce the structure and functions of natural DNA/RNA oligomers, designed to increase the stability, binding affinity and specificity of the corresponding oligonucleotide sequences through chemical modifications. ASOs can induce targeted degradation of mRNA, usually through an RNase-H‐dependent mechanism (**Figure 1**). Splice-modulating ASOs have been developed to treat inborn errors of metabolism by targeting abnormal splicing caused by mutations, thereby restoring the expression of normal transcripts and eliminating the deficiency of functional proteins (14).

RNA-targeted therapy can precisely modulate the function of target RNA with minimal off-target effects and can be rationally designed based on sequence data. These oligonucleotide drugs can be either single-stranded or double-stranded. Unlike double-stranded siRNAs, which destroy only target RNA to reduce gene expression, ASOs have greater versatility; they can degrade RNA, regulate gene expression or process mRNAs through various mechanisms. In addition, ASOs allow a wider range of chemical modifications than siRNA (15).

**Figure 2:**
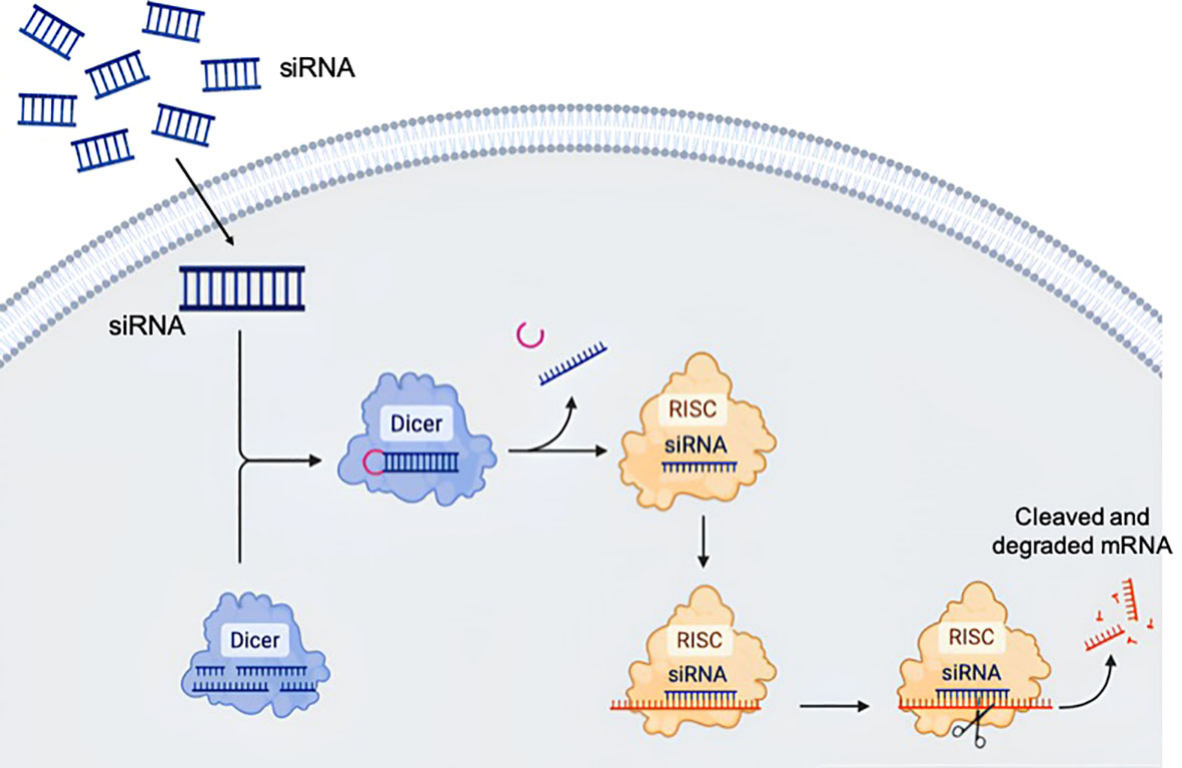
Biological principle of RNA interference. The long double stranded RNA is transferred by endocytosis to the cytosol and fragmented by the Dicer protein into short duplexes. The resulting nucleic acid is called small interfering ribonucleic acid (siRNA). siRNA is incorporated into the RNA-induced silencing complex (RISC), where the siRNA gets unwound. In this process, the RISC selects one strand as the guide or antisense strand, while the complementary passenger strand is degraded. The guide strand can bind the target sequence and alter gene expression. The drawing was created using BioRender web-tool.

RNA interference is an internal mechanism of post-transcriptional gene regulation that has been used to develop therapy since the discovery by Andrew Fire, Craig Mello and colleagues in 1998 that double-stranded RNAs can induce RNA interference to catalyze the degradation of complementary mRNA transcripts in *Caenorhabditis elegans* (16). RNA interference-based drugs are small, non-coding double-stranded RNA molecules consisting of 21–23 nucleotides, that are designed to target specific mRNAs and induce gene silencing through a complex intracellular system known as the RNA-induced silencing complex (RISC). In the cellular environment double-stranded siRNA integrates into RISC, which contains many proteins. The siRNA gets unwound and separated into semantic and antisense strands, and only the antisense strand remaining in the complex. This antisense strand subsequently binds to its complementary mRNA target, promoting cleavage and leading to the degradation of the RNA molecule (**Figure 2**) (17, 18).

Two decades passed since the concept of ASOs and RNA interference mechanisms appeared before RNA therapy found clinical use, respectively. The Food and Drug Administration (FDA) approved the first ASO drug Formivirsen for CMV retinitis in 1998, and in 2018 – the first siRNA drug Patisiran for the treatment of polyneuropathy caused by hereditary transthyretin-mediated amyloidosis (7). Despite the successful use of RNA interference and ASOs in basic science to influence the expression of target genes, there have been few successful attempts to use them in the treatment of diseases. The most important and still unresolved problem is the efficient delivery of antisense molecules to target cells *in vivo*.

Scientists are currently actively researching the use of antisense therapy to treat a wide range of diseases, including type 2 diabetes (19). In this review we decided to focus on diseases of the endocrine system. The purpose of this review is to discuss potential RNA-targeted therapeutic opportunities in diabetes mellitus, including monogenic beta cell diabetes, and obesity, including syndrome obesity and monogenic obesity, as well as in non-monogenic or complex endocrine disorders, rare and ultrarare endocrine diseases.

## Potential therapeutic intervention for polygenic or complex endocrine and metabolic diseases

### Diabetes mellitus

Diabetes mellitus is a metabolic disorder characterized by elevated blood glucose levels and associated symptoms. This complex condition includes immune-mediated type 1 diabetes, specific types of diabetes due to other causes, genetically determined diabetes, and widespread type 2 diabetes. According to recent data from the International Diabetes Federation, almost 240 million of the patients with type 2 diabetes mellitus are unaware of their condition and therefore undiagnosed, while 537 million individuals were diagnosed with diabetes in 2021, the number of which is expected to increase to 643 million by 2030 and 783 million by 2045 (20).

In the past decade, genome-wide association studies (GWAS) and several large multicenter research studies have greatly enhanced our understanding of the genetic basis of type 1 diabetes mellitus. More than 75 genetic loci related to type 1 diabetes mellitus have been identified through GWAS, including variations in HLA alleles (21, 22). The identification of target genes may be more important for drug development and therapeutic interventions than for assessing the risk of developing type 1 diabetes.

Type 2 diabetes, known as non-insulin-dependent diabetes, has a complex polygenic and multifactorial origin. Its treatment involves various classes of oral hypoglycemic agents such as biguanides, thiazolidinediones, sulfonylureas, meglitinides, dipeptidyl peptidase-4 inhibitors, glucagon-like peptide-1 receptor agonists, a-glucosidase inhibitors, sodium glucose cotransporter-2 inhibitors. However, these medications do not prevent the progression of type 2 diabetes mellitus and are unable to influence the transcriptional or translational processes of diabetes-related genes, demonstrating less efficacy compared to RNA-targeting interventions.

Antisense-mediated gene knockdown by specific modification of target gene expression involved in pathogenesis of type 2 diabetes mellitus gives new hope for the development of antisense-based therapy. In this section, we will focus only on potential therapeutic molecules, targeting which with ASOs can slow the development and progression of type 2 diabetes mellitus.

Pancreatic beta cells represent a promising target for therapeutic interventions using ASOs, due to their critical role in both type 1 and type 2 diabetes mellitus. Ämmälä et al. (23) demonstrated the ability of ASOs conjugated with glucagon-like peptide 1 receptor agonists to effectively reach beta cells both *in vitro* and *in vivo*. However, the problem of extrahepatic delivery of ASOs limits the wider use of this class of RNA-based therapeutic agents.

Both β-cells and gastrointestinal enteroendocrine cells express a diverse range of G protein-coupled receptors (GPCRs) that regulate insulin secretion, which requires significant research and development to create therapeutic drugs targeting these receptors. GPCRs can control glucose homeostasis through modulation of insulin and incretin secretion, with potential targets for diabetes treatment including free fatty acid receptor 1 (FFAR1), also known as G-protein coupled receptor 40 (GPR40), free fatty acid receptor 4 (FFA4, also known as GPR120), and glucose-dependent insulinotropic receptor (GPR119) (24). ASOs targeting glucagon receptor expression have been shown to reduce diabetic symptoms in *db/db* mice (25).

Sloop et al. (26) targeted the glucagon receptor (GCGR) in rodent models of type 2 diabetes. Treatment with GCGR ASOs led to decreased GCGR expression, normalization of blood glucose levels, improved glucose tolerance and preserved insulin secretion. Furthermore, GCGR inhibition resulted in increased serum levels of active glucagon-like peptide-1 (GLP-1) and insulin in pancreatic islets. These findings suggest that targeting GCGR could offer clinical benefits for type 2 diabetes mellitus by reducing glucose production and improving pancreatic beta-cell function.

Another potential target is Protein Tyrosine Phosphatase 1B (PTP1B), an intracellular protein tyrosine phosphatase expressed in various cells and tissues, with its encoding gene *PTPN1* located in a region linked to insulin resistance and obesity. PTP1B plays an important role in the regulation of insulin metabolic pathways (27). PTP1B dephosphorylates the insulin receptor as well as insulin receptor substrates proteins (28).

Evidence of the importance of PTP1B in glucose metabolism was obtained from studies on PTP1B knock-out mice, which exhibited increased tyrosine phosphorylation of the insulin receptor in the liver, along with reduced blood glucose and insulin levels compared to wild-type mice (29). PTP-1B-deficient (PTP-1B^−/−^) mice also demonstrated increased insulin sensitivity and resistance to weight gain when subjected to a high-fat diet (30).

Additionally, targeting PTP1B improved glycemic control in obese and insulin-resistant diabetic *ob/ob* and *db/db* mice, positively impacting the expression of insulin signaling proteins in the liver and adipose tissue, thereby improving insulin sensitivity in liver (31). Consequently, inhibiting PTP1B presents a potential therapeutic strategy for diabetes. Despite improvement strategies continue to be proposed, the only PTP1B inhibitors that have progressed to clinical trials, such as ertiprotafib (a non-competitive pleiotropic inhibitor), ISIS 113715 and ISIS 404173 (ASO inhibitors), trodusquemine (an allosteric inhibitor), and JTT-551 (a hybrid inhibitor), all the trials were discontinued due to adverse side effects and low specificity (32, 33).

Forkhead box O (Foxo)-1 is very important in glucose metabolism as it stands out as an attractive target for pharmacological intervention. Fasting hyperglycemia is mainly due to an increased gluconeogenesis, with Foxo1 recognized as a key transcription factor in this process. Foxo1 regulates the expression of several genes, including pancreatic duodenal homeobox-1 in β-cells, pyruvate dehydrogenase kinase 4, lipoprotein lipase, adiponectin receptors in muscle, and PPAR-γ and GLUT4 in adipocytes (34). In diabetic *db/db* mice, targeting Foxo1 led to a decrease in the transcriptional expression of genes encoding gluconeogenic enzymes, and, as a result, reduced the blood glucose levels (35).

It is assumed that a decrease in Foxo1 expression leads to a decrease in hepatic gluconeogenesis, increases hepatic insulin action and stimulates an improved peripheral insulin-stimulated glucose metabolism.

Research by Nakae et al. (36) involving Foxo1 haploinsufficient mice crossed with insulin receptor-deficient mice, along with studies by Altomonte et al. (37), in which *db/db* mice treated with an adenovirus expressing a dominant-negative Foxo1 mutant, demonstrated that reduced Foxo1 activity resulted in decreased levels of key gluconeogenic enzymes mRNA and lowered blood glucose concentrations.

ASO therapy aimed at reducing the level of Foxo1 mRNA in mouse hepatocytes effectively decreased both the expression of Foxo1 protein and mRNA. In mice with obesity and insulin resistance, Foxo1 ASO-therapy resulted in lower plasma glucose levels and reduced endogenous glucose production (38). Thus, targeting FoxO1 and developing strategies for tissue-specific delivery of FoxO1 therapeutics can serve as an effective approach for treating type 2 diabetes mellitus and improving insulin resistance.

Recent studies have also shown that metastasis-associated lung adenocarcinoma transcript 1 (MALAT1) plays a crucial role in various pathophysiological processes, including the progression of diabetes and diabetic-related complications, by influencing gene transcription. MALAT1 can potentially serve as a new biomarker and therapeutic target for the treatment of complications associated with diabetes mellitus. Moreover, therapeutic strategies targeting MALAT1 may provide promising options for the prevention and treatment of diabetes-related diseases.

The expression of MALAT1 is elevated in different diabetic-related complications including cerebral ischemic reperfusion injury-induced by diabetes mellitus, diabetic retinopathy, diabetic cataract, atherosclerosis, diabetic cardiomyopathy, diabetic gastropathy, diabetic kidney disease and gestational diabetes mellitus (39). Overexpression of MALAT1 indicates an important pathogenetic mechanism of microvascular dysfunction associated with diabetes – hyperproliferation of endothelial cells through p38MAPK signaling. Inhibition of MALAT1 may become a potent antiangiogenic therapy for the treatment of diabetic microvascular complications (40). In general, elevated levels of MALAT1 expression in various complications associated with diabetes mellitus, as well as therapeutic effects on MALAT1 with synthetic oligonucleotides and siRNAs, define MALAT1 as a therapeutic target and potential biomarker.

Various pathogenetic factors and mechanisms interact and lead to the development and progression of diabetes mellitus. Currently, diabetes management strategies primarily focus on improving glycemic control and increasing tissue sensitivity to insulin, as well as preventing macro- and microvascular complications and reducing their severity. Therefore, it is necessary to search for new therapeutic agents for the treatment of diabetes and its related complications.

### Polygenic obesity

Obesity has become a worldwide health problem due to its increasing prevalence and comorbidities. The World Health Organization (WHO) defines overweight and obesity as abnormal or excessive fat accumulation that presents a risk to health (41). According to recent data, the global age-standardized prevalence of obesity has increased from 8.8% (8.5–9.1) in 1990 to 18.5% (17.9–19.1) in 2022 in women and from 4.8% (4.6–5.0) to 14.0% (13.4–14.6) in men. The number of obese women and men in 2022 was 504 million (489–520) and 374 million (358–391), respectively, which was an increase of 377 million (360–393) and 307 million (290–324), respectively, from 1990 (42).

The prevalence of pediatric obesity has increased worldwide over the past five decades. In 2019 the World Obesity Federation estimated that 206 million children and adolescents between the ages of 5 and 19 will be obese in 2025, and 254 million in 2030 (43). Obese children have an increased risk of various medical health problems, which makes early diagnosis and intervention extremely important; however, currently available pharmacological options are limited.

The pathophysiology of obesity involves dysfunction in the main regulatory pathways of energy balance, which are influenced by both genetic and environmental factors (44). Obesity can be classified into polygenic obesity, monogenic obesity, and obesity syndrome based on genetic characteristics. The most common form of obesity is polygenic obesity, which results from the cumulative effects of multiple genetic factors combined with environmental influences (45). In contrast, monogenic and syndromic obesities are caused by rare genetic variants affecting as few as one gene and/or deletions of chromosomal regions containing genes, involved in key obesity pathogenesis pathways, leading to early-onset and severe obesity (46).

Polygenic variants associated with obesity are common in general population, with allele frequencies greater than 1%, but their individual effect sizes are relatively small (47). In rare cases, inheritance of obesity may be explained by a large-effect mutation that disrupts energy homeostasis or fat deposition. However, for most severely obese individuals, genetic predisposition is likely due to the cumulative effects of multiple variants with an individual moderate effect – a «polygenic» model (48). This paradigm is similar to other complex diseases in which polygenic inheritance, including many common genetic variants, makes up a significant part of the hereditary predisposition.

A recent genome-wide association study (GWAS) quantified the relationship between 2.1 million common genetic variants and body mass index in 339 224 individuals, identified 97 genome-wide loci associated with body mass index. These loci accounts for 2.7% of the variation in body mass index, and suggests that as much as 21% of body mass index variation can be explained by general genetic variation (49). Specific genes that may be associated with body mass index have been identified. Many of these genes are associated with processes in the central nervous system, including synaptic function, intercellular adhesion, and glutamate signaling (*CADM2*, *ELAVL4*, *GRID1*, *NRXN3*, *NEGR1*, *SCG3*), cause monogenic obesity syndromes (*BDNF*, *BBS4*, *MC4R*, *POMC*), or function in extreme/early onset obesity in humans and mouse models (*NEGR1*, *SH2B1*). Other genes are related to insulin secretion and action, energy metabolism, lipid biology and/or adipogenesis (*APOBR*, *ASB4*, *CREB1*, *FAM57B*, *FOXO3*, *GIPR*, *HSD17B12*, *IRS1*, *NPC1*, *RPTOR*, *TCF7L2*), encode RNA binding/processing proteins (*CELF1*, *ELAVL4*, *PTBP2*, *RALYL*), participate in the MAP kinase signaling pathway (*MAP2K5* and *MAPK3*) or regulate cell proliferation or cell survival (*FAIM2*, *OLFM4*, *PARK2*) (49).

Genetic risk predictors are important for clinical medicine because they identify people at risk before the disease manifests itself. GWAS identified about 1000 nearly independent single nucleotide polymorphisms (SNPs) associated with body mass index, which explain about 6% of the body mass index variance (50). However, for most of these loci the specific causal genes, their functional roles in various cells, tissues and organs regarding body weight, and the underlying mechanisms remain unknown.

The main cause of polygenic obesity and overweight is an imbalance between energy intake and energy expenditure. Therapeutic strategies that increase energy expenditure or maintain basal energy expenditure during caloric deficits can be highly effective. The growing number of treatment options and technological advances have led to increasing interest in personalizing obesity treatment to maximize benefits and safety. Consequently, using ASOs to target genes that regulate metabolism may provide a therapeutic opportunity to increase peripheral energy expenditure.

An illustrative example is fibroblast growth factor receptor 4 (FGFR4) and its ligand, fibroblast growth factor (FGF15) in rodents, or its human and primates ortholog FGF19. FGFR4 is predominantly expressed in the hepatocytes and some other peripheral tissues, while its expression in adipose tissue is minimal, and absent in heart tissue. Besides regulating bile acid metabolism, FGFR4 is also involved in lipid, carbohydrate and energy metabolism. The expression of FGFR4 in liver decreases with fasting and increased by insulin (51). Administration of FGF19 to obese mice or its overexpression has been reported to increase metabolic rate and improve obesity, hepatic steatosis, insulin sensitivity and plasma lipid levels (52, 53). FGF19 has also been found to inhibit fatty acid synthesis in the liver (54), stimulate glycogen synthesis (55) and reduce hepatic gluconeogenesis (56).

Yu et al. (57) reported that FGFR4 specific ASO treatment of mice with diet-induced obesity resulted in decreased FGFR4 expression in liver, leading to reduced body weight of obese mice by more than 20% and adiposity, as well as improved insulin sensitivity and liver steatosis. The weight loss associated with antisense therapy was due to a decrease in body fat. Histological examination with hematoxylin and eosin staining showed a decrease in the size of adipocytes in adipose tissue of mice treated with FGFR4 ASO, without redistribution of triglycerides to either the liver or muscles. This FGFR4 ASO anti-obesity effect persisted in animals with limited calorie intake. An antisense decrease in FGFR4 expression resulted in elevated plasma FGF15 levels, increased fatty acid oxidation rates in tissues, enhanced overall metabolic rates, and decreased tissue lipogenesis in obese mice. This study demonstrates that FGFR4 inhibition may be a potential therapeutic approach for the treatment of obesity and related metabolic disorders.

A better understanding of the key regulators and pathogenetic processes involved in the development and progression of obesity also suggests the possibility of ASOs influence on targeting components of the enzymatic pathway for converting dietary carbohydrate into fat, or *de novo* lipogenesis pathway, such as stearoyl–CoA desaturase-1 (58).

Another example of the use of ASOs in the context of metabolic syndrome involves targeting ANGPTL8, which regulates the expression of lipoprotein lipase (LPL). ANGPTL8 is an LPL inhibitor that is expressed strongly in the white adipose tissue (59). Vatner et al. (60) demonstrated that pharmacological inhibition using ASO against *Angptl8* in adult high-fat-fed rodents improves adipose function, increasing postprandial triacylglycerol uptake and preventing ectopic lipid accumulation and lipid-induced insulin resistance.

Antisense inhibition of monoacylglycerol acyltransferase 1, which catalyzes the formation of mono- to diacylglycerols in the lipid synthesis pathway, and targeting with ASOs specific to diacylglycerol O-Acyltransferase 2, catalyzes the final stage in the triacylglycerol synthesis pathway, are also probably promising targets for the treatment of impaired regulation of lipid homeostasis in the setting of obesity and its complications (58).

Therapeutic strategies based on siRNA suppression of pro-adipogenic genes present a novel approach for localized fat reduction. Among the factors associated with persistent fat deposits, transforming growth factor β1 (TGF-β1) and cyclooxygenase 2 (COX-2) are promising target candidates. TGF-β1 is a cytokine that plays a crucial role in regulating adipogenesis and fat distribution, while COX-2 is an enzyme involved in adipocyte differentiation and is highly expressed in metabolically active fat cells (61). Employing siRNA to inhibit the expression of TGF-β1 and COX-2 in stubborn fat deposits could disrupt their metabolic functions and induce apoptosis, leading to targeted fat reduction. Preclinical *in vitro* and animal model studies of an injectable TGF-β1 and COX-2 siRNAs encapsulated in a polypeptide nanoparticle, have demonstrated its safety and tolerability, substantiated its mechanisms, while offering initial evidence of its effectiveness in reducing fat (62). By highlighting the potential of siRNA to target key genes involved in adiposity, these preclinical studies pave the way for developing innovative fat reduction therapies and the progression to clinical trials.

Future clinical research should focus on verifying the safety and efficacy of siRNA-based treatments in human populations, considering factors such as individual genetic variability, the durability of gene silencing effects and the risk of immune responses. Additionally, optimizing delivery systems and refining dosing strategies will be essential to improve the clinical applicability and effectiveness of siRNA-based treatments.

## Possibilities of ASO therapies for the treatment of monogenic and syndromic disorders

### Future drug discovery perspectives for monogenic diabetes

Diabetes caused by monogenic alterations, which represents approximately 1–5% of cases in pediatric and young populations (63, 64), is more suitable to targeted therapies. Monogenic diabetes results from defects in a single gene or in chromosomal locus and is classified into neonatal or early infancy diabetes, maturity-onset diabetes of the young (MODY), diabetes associated with extra-pancreatic features, and monogenic insulin resistance syndromes (65). Most cases of monogenic diabetes are caused by mutations in genes that control beta cell-function. Different types of monogenic diabetes are summarized in **Tables 1** and **2**. A deep understanding of the genetic basis of this disease contributes to the development of targeted treatment methods aim to address the primary cause of the disease.

**Table 1:** Monogenic subtypes of neonatal and infancy‐onset diabetes (65).

Subtype of monogenic diabetes	Gene symbol	Gene full name	Locus	Inheritance
Abnormal pancreatic development				
Transient neonatal diabetes	*HNF1B*	Hepatocyte nuclear factor 1 homeobox B	17q21.3	Dominant
	*PLAGL1/HYMAI*	Pleomorphic adenoma gene like zinc finger 1/Hydatidiform mole associated and imprinted	6q24	Variable (imprinting)
	*ZFP57*	Zinc finger protein 57 homolog	6p22.1	Recessive
Permanent neonatal diabetes	*CNOT1*	Carbon catabolite repression 4-negative on TATA-less transcription complex subunit 1	16q21	Spontaneous
	*GATA4*	GATA binding protein 4	8p23.1	Dominant
	*GATA6*	GATA-binding factor 6	18q11.1–q11.2	Dominant
	*GLIS3*	Transcription factor Gli-similar 3	9p24.3–p23	Recessive
	*MNX1*	Motor neuron and pancreas homeobox 1	7q36.3	Recessive
	*NEUROD1*	Neurogenic differentiation 1	2q32	Recessive
	*NEUROG3*	Neurogenin 3	10q21.3	Recessive
	*NKX2-2*	Homeodomain protein NK2 homeobox 2	20p11.22	Recessive
	*ONECUT1*	One cut homeobox 1	15q21.3	Recessive
	*PAX6*	Paired box protein	11p13	Recessive
	*PDX1*	Pancreatic and duodenal homeobox 1	13q12.1	Recessive
	*PTF1A*	Pancreas associated transcription factor 1a	10p12.2	Recessive
	*PTF1A enhancer*	Pancreas associated transcription factor 1a -specific enhancer	10p12.2	Recessive
	*RFX6*	Regulatory factor X6	6q22.1	Recessive
Abnormal β-cell function				
Transient neonatal diabetes/Permanent neonatal diabetes	*ABCC8*	ATP-binding cassette C8	11p15.1	Spontaneous, dominant or recessive
Permanent neonatal diabetes	*GCK*	Glucokinase	7p15–p13	Recessive
Permanent neonatal diabetes or transient neonatal diabetes	*INS*	Insulin	11p15.5	Recessive
Permanent neonatal diabetes	*KCNMA1*	Potassium calcium-activated channel subfamily M alpha 1	10q22.3	Spontaneous
Permanent neonatal diabetes/Transient neonatal diabetes	*KCNJ11*	Potassium inwardly rectifying channel subfamily J member 11	11p15.1	Spontaneous or dominant
Permanent neonatal diabetes (Fanconi-Bickel syndrome)	*SLC2A2 (GLUT2)*	Solute carrier family 2 member 2 (Glucose transporter 2)	3q26.1–q26.3	Recessive
Permanent neonatal diabetes (Roger’s syndrome)	*SLC19A2*	Solute carrier family 19 member 2	1q23.3	Recessive
Destruction of β cells				
Permanent neonatal diabetes	*EIF2B1*	Eukaryotic translation initiation factor 2B subunit alpha	12q24.31	Spontaneous
	*IER3IP1*	Immediate early response 3 interacting protein 1	18q21.2	Recessive
	*INS*	Insulin	11p15.5	Spontaneous or dominant
	*ITCH*	Itchy E3 ubiquitin protein ligase	20q11.22	Recessive
	*LRBA*	LPS responsive beige-like anchor protein	4q31.3	Recessive
	*STAT3*	Signal transducer and activator of transcription 3	17q21.2	Spontaneous
	*YIPF5*	Yip1 domain family member 5	5q31.3	Recessive
	*WFS1*	Wolframin ER transmembrane glycoprotein	4p16.1	Recessive
Permanent neonatal diabetes or infancy-onset diabetes	*WFS1*		4p16.1	Dominant
Permanent neonatal diabetes (Wolcott-Rallison syndrome)	*EIF2AK3*	Eukaryotic translation initiation factor 2-alpha kinase 3	2p11.2	Recessive
Autoimmune lymphoproliferative syndrome which can include autoimmune diabetes	*CTLA4*	Cytotoxic T-lymphocyte associated protein 4	2q33.2	Spontaneous
Immunodeficiency-41 syndrome (complex disorder of immune dysregulation)	*IL2RA*	Interleukin 2 receptor subunit alpha	10p15.1	Recessive
IPEX syndrome (X-linked immune dysregulation, polyendocrinopathy, enteropathy)	*FOXP3*	Forkhead box P3	Xp11.23-p13.3	X-linked, recessive

**Table 2:** Monogenic forms of diabetes mellitus type MODY.

MODY variant	OMIM	Locus	Responsible gene	Gene full name	Gene function	Relative prevalence
MODY 1	125850	20q13.12	*HNF4A*	Hepatocyte nuclear factor 4-alpha	Transcription factor	3–5% of MODY
MODY 2	125851	7p15–p13	*GCK*	Glucokinase	Enzyme in the first step of glucose metabolism	30–70% of MODY
MODY 3	600496	12q24	*HNF1A*	Hepatocyte nuclear factor 1-alpha	Transcription factor	30–50% of MODY
MODY 4	606392	13q12.1	*PDX1*	Pancreas/duodenum homeobox protein-1	Transcription factor	< 1% of MODY
MODY 5	137920	17q21.3	*HNF1B*	Hepatocyte nuclear factor 1-beta	Transcription factor	3–5% of MODY
MODY 6	606394	2q31.3	*NEUROD1*	Neurogenic differentiation factor 1	Transcription factor	Very rare
MODY 7	610508	2p25.1	*KLF11*	Krüpell-like factor 11	Transcription factor	Very rare
MODY 8	609812	9q34.13	*CEL*	Cholesteryl-ester lipase	Controls exocrine and endocrine functions of pancreas	Very rare
MODY 9	612225	7q32.1	*PAX4*	Paired homeobox 4	Transcription factor	Very rare
MODY 10	613370	11p15.5	*INS*	Insulin	Encode the proinsulin precursor	< 1% of MODY
MODY 11	613375	8p23.1	*BLK*	B-lymphoid tyrosine kinase	Tyrosine kinase functions in signal transduction	Very rare
MODY 12	600509	11p15.1	*ABCC8*	ATP-binding cassette C8	Regulating insulin release	< 1% of MODY
MODY 13	616329	11p15.1	*KCNJ11*	Inward-rectifying potassium channel J11	Regulating insulin release	< 1% of MODY
MODY 14	616511	3p14.3	*APPL1*	Adaptor protein, phosphotyrosine interaction, PH Domain, and leucine zipper-containing 1	Insulin signal pathway	Very rare

Monogenic β-cell dysfunction, known as MODY, was first clinically recognized in the 1970s. MODY accounts for 1–6% of all diabetes cases (63, 66, 67). After the identification of the first genetic mutations in the 1990s regarding *GCK* (MODY2), *HNF1A* (MODY3) and *HFN4A* (MODY1), advances in DNA sequencing methods techniques have significantly improved the discovery of new causal variant. To date, at least 14 distinct MODY subtypes (**Table 2**) have been identified, each associated with single-gene mutations inherited by autosomal dominant pattern, which play key roles in the differentiation, development and function of β-cells (68, 69). The recent application of advanced DNA sequencing techniques has led to the discovery of new genes in MODY cases such as *AKT2*, *CACNA1E*, *EIF2AK3*, *GLIS3*, *HADH, MNX1*, *MTOR*, *NEUROG3*, *NKX2.2*, *NKX6.1*, *PCBD1*, *PTF1A*, *RFX6*, *TBC1D4* and *WFS1* (70). In addition, there is a high prevalence ranging from 46.2 to 73.9% of so-called MODY-X patients who meet the classic MODY diagnostic criteria but do not have a specific genetic diagnosis (71).

Personalized treatment approaches for monogenic diabetes are being advanced due to a deeper understanding of its genetic basis.

Monogenic diabetes resulting from mutations in genes that encode the insulin receptor, leads to alterations in its biosynthesis and post-translational processing. This results in receptor degradation and a reduction in insulin binding or receptor activation. This form of diabetes is associated with various syndromes of generalized insulin resistance, often severe, and is characterized by short stature and dysmorphic features, as seen in Donohue syndrome and Rabson-Mendenhall syndrome. Such syndromes typically arise from homozygous or compound heterozygous mutations. A milder form associated with the insulin receptor gene is type A insulin resistance, which can be inherited in both dominant and recessive patterns (65, 72).

The advent of molecular therapy offers new possibilities, as targeted interventions aimed at specific genetic defects can significantly change the management of monogenic diabetes. Development of new agents-especially antisense RNA will provide additional management options.

### Gene-based therapy as a strategy to fight monogenic and syndromic obesities

Rare monogenic obesity is mainly associated with single gene variants within the hypothalamic MC4R pathway, while syndromic obesity is characterized by severe obesity accompanied by intellectual disabilities, dysmorphic features, systemic abnormalities, and characterized by low frequency, high variability and Mendelian inheritance pattern (73).

Monogenic obesity mainly occurs as a result of mutations in the genes of the leptin-melanocortin pathway that regulate food intake (genes of leptin (*LEP*) and leptin receptor (*LEPR*), proopiomelanocortin (*POMC*), proconvertase 1 (*PC1*)), or in specific genes associated with these pathways (73). The most frequent forms of syndromic obesity are Prader-Willi syndrome, Bardet-Biedle syndrome and Alstrom syndrome.

Clinical and genetic characteristics of monogenic and syndromic obesity diseases are presented in **Table 3**. Patients with monogenic or syndromic obesities usually experience early onset of severe obesity and hyperphagia.

**Table 3:** Genetic and clinical characteristics of main monogenic forms of obesity and rare syndromic forms of obesity.

Gene or Syndrome	Inheritance	Prevalence	Clinical features and endocrine abnormalities
Monogenic forms of obesity			
MCR4 deficiency	Autosomal dominant or autosomal recessive	2–5% of patients with severe early-onset obesity	Hyperphagia, extreme early-onset obesity, severe hyperinsulinemia, increased lean body mass, accelerated linear growth
Leptin deficiency	Autosomal recessive	Diagnosed in fewer than 100 patients worldwide	Severe hyperphagia, early-onset obesity, frequent infections, hypogonadotropic hypogonadism, hypothyroidism
Leptin receptor deficiency	Autosomal recessive	2–3% of patients with severe early-onset obesity	Hyperphagia, impaired satiety, rapid weight gain, severe obesity, recurrent infections, hypogonadotropic hypogonadism, hypothyroidism
POMC deficiency	Autosomal recessive	Diagnosed in fewer than 10 patients worldwide	Hyperphagia, severe early-onset obesity, adrenal insufficiency, pigmentary abnormalities including pale skin or red hair
PCSK1 deficiency	Autosomal dominant or autosomal recessive	Diagnosed in fewer than 20 patients worldwide	Severe malabsorptive diarrhea in the neonatal period, postprandial hypoglycemia, early-onset obesity, hypogonadotropic hypogonadism, diabetes insipidus, hypothyroidism, adrenal insufficiency
SH2B1 deficiency	Autosomal recessive	Not reported	Hyperphagia, early-onset obesity, severe insulin resistance, maladaptive behaviors, reduced adult height
NCOA1 (also known as SRC1) deficiency	Autosomal recessive	Not reported	Hyperphagia in childhood, severe obesity, multiple fractures with minimal trauma starting from childhood, persistent diarrhea, partial thyroid hormone resistance, menorrhagia
SIM1 deficiency	Not reported	Diagnosed in fewer than 50 patients worldwide	Neonatal hypotonia, feeding difficulty, developmental delay, facial dysmorphism, hypogonadism
NTRK2 deficiency	Not reported	Diagnosed in fewer than 10 patients worldwide	Severe obesity from the first months of life, developmental delay, behavioral disturbance, blunted response to pain
Syndromic forms of obesity			
Prader-Willi syndrome	Varies	1/15 000 to 1/20 000 births	Neonatal hypotonia, hyperphagia, obesity, abnormal body composition with increased fat mass and reduced lean body mass, hypogonadotropic hypogonadism, growth hormone deficiency, intellectual disability, learning difficulties, behavioral problems
Bardet-Biedle syndrome	Autosomal recessive	1/125 000 to 1/175 000 births	Early-onset obesity, retinal dystrophy, polydactyly, kidney abnormalities, hypogonadism, learning difficulties
Alstrom syndrome (*ALMS1*)	Autosomal recessive	Diagnosed in about 950 patients worldwide	Obesity, cone-rod dystrophy, progressive bilateral sensorineural hearing loss, cardiomyopathy, insulin resistance, chronic progressive kidney disease
Albright hereditary osteodystrophy (*GNAS1*)	Autosomal dominant	1/1 000 000 births	Early-onset obesity, short stature, round facies, shortening of 4th and/or 5th metacarpal and metatarsal bones, subcutaneous ossification, pseudohypoparathyroidism
Börjeson-Forssman-Lehmann syndrome (*PHF6*)	X-linked, recessively inherited	Approximately 50 reported patients	Obesity, mental retardation, dysmorphic features, epilepsy, hypogonadism
Cohen (*COH1*)	Autosomal recessive	Diagnosed in fewer than 1000 patients worldwide	Obesity, intellectual disability, distinct craniofacial abnormalities, intellectual disability, myopia, hypotonia, and skeletal malformations
16p11.2 deletion syndrome	Autosomal dominant	Approximately 3/10 000 births	Developmental delay, intellectual disability, communication and socialization difficulties, hyperinsulinemia

In contrast to polygenic obesity, knowing the gene that causes monogenetic obesity can help guide treatment. Over the past ten years, genetic analysis has made it possible to develop individualized treatment options for some types of monogenic obesity.

Patients with monogenic and syndromic obesity may benefit from new gene technologies. This approach may be considered in patients who are not suitable for pharmacotherapy or have failed other treatments. There are several studies that focused on targeting adipose tissue using adeno-associated viruses and injecting the virus into visceral or subcutaneous adipose in obese mice (74, 75).

O’Neill et al. (76) developed an *in vivo*, systemic method for gene transfer that specifically targets adipose tissue of *ob/ob* mice through the use of adeno-associated virus vectors. The specificity for adipose tissue was increased by inserting synthetic microRNA-122 (miR-122) target sites into the 3’ untranslated region (UTR) of the expression cassette. This study demonstrated stable and largely selective gene transfer to several adipose tissue depots following a single systemically administration of the gene expression vector. The researchers replaced the enhanced green fluorescent protein (eGFR) gene in the adeno-associated virus2/8-Adipo-eGFP_miR122_ vector with human leptin (adeno-associated virus2/8-Adipo-Leptin_miR122_) and injected it into the tail vein of nine-week-old *ob/ob* mice with leptin deficiency. The *ob/ob* mouse with complete absence of leptin protein exhibits phenotype remarkably similar to congenital leptin deficiency in human, and therefore represents an effective model for human disease to test the therapeutic potential of adipose targeted adeno-associated virus vector. Control *ob/ob* mice were injected with adeno-associated virus2/8-Adipo-eGFP_miR122_. After 4–7 days post-virus administration, the leptin-treated group already consumed less food and lost weight gradually than control *ob/ob* mice.

The ability to replace a defective protein secreted by adipose tissue and correct physiological defect using an adipose-targeted adeno-associated virus vector in a rodent model of human disease is promising for therapeutic applications. Currently, there are no published data on the development of ASOs that specifically affect adipocytes. Moreover, comprehensive preclinical and clinical studies are needed to apply these new therapeutic methods in clinical practice.

## Gene-based therapy for rare and ultrarare diseases: a perspective of patient-customized N-of-1 approach

Rare diseases are numerous, geographically disparate and heterogeneous in nature. A limited number are preventable or curable; most are chronic and many lead to premature mortality. In Europe rare diseases are defined by an incidence of 1 in ≤ 2,000. There are an estimated 6.000–8.000 rare diseases and ∼80% of those have a genetic cause (77, 78). Furthermore, the global point prevalence of rare diseases was calculated as 3 482.3–5 910.3 per 100 000 (~3.5–5.9%) in the general population (77). According to this forecast, from 17.8 to 30.3 million people in the European Union and from 262.9 to 446.2 million people worldwide are affected by a rare disease (77).

While some rare diseases are relatively more common with incidences ranging from 1:2000 to 1:10000, the majority of them are classified as «ultrarare» and occur in less than 1 in 100000 individuals (77). However, there is no formal definition for «ultrarare» disease; this subcategory was informally introduced by the National Institute for Health and Care Excellence for drugs intended for diseases with a prevalence of less than 1 per 50000 individuals (79).

**Figure 3:**
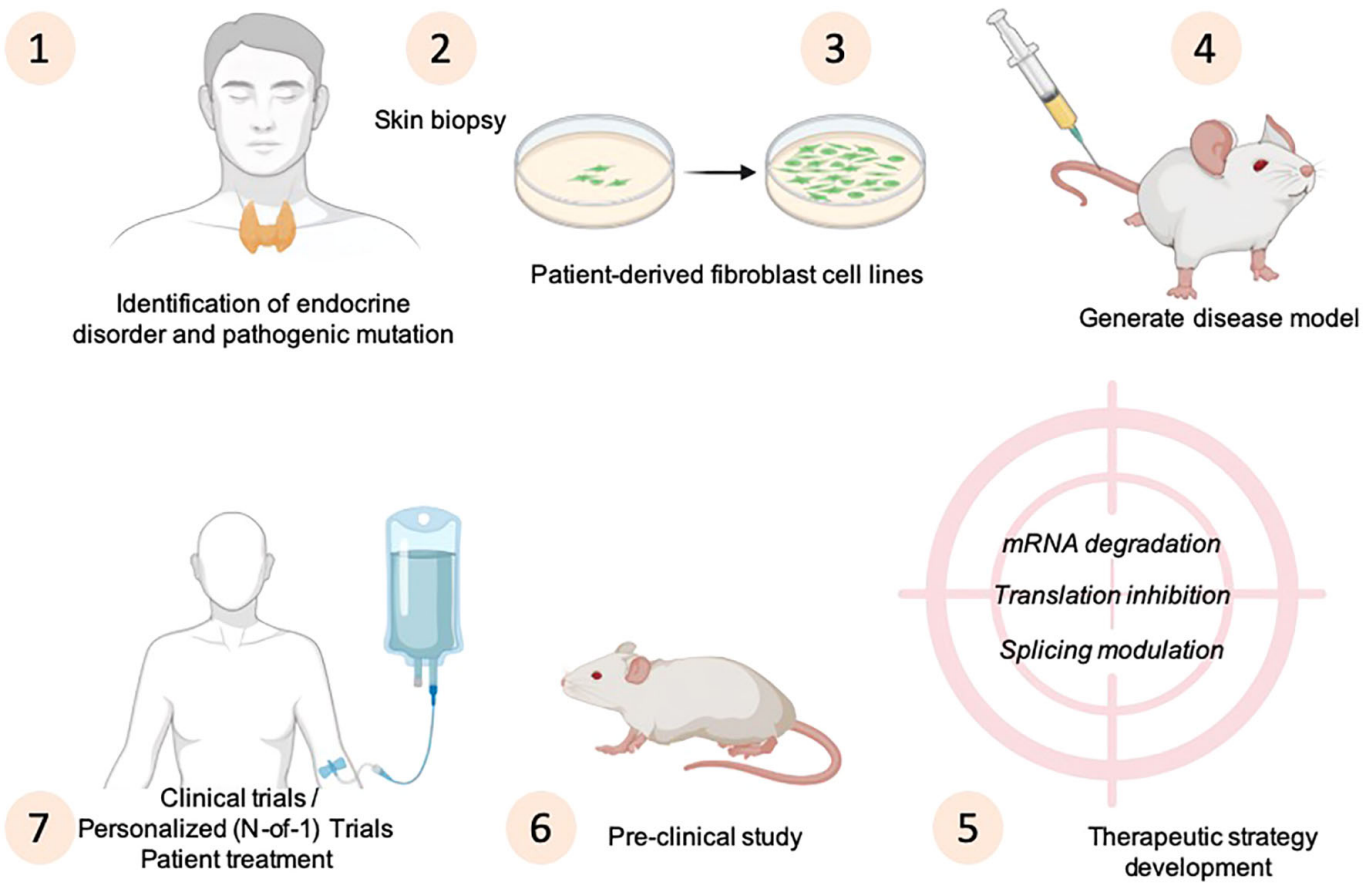
Strategies for disease modeling and principles of N-of-1 designs to develop personalized medicine approaches for endocrine disorders. The drawing was created using BioRender web-tool.

Due to the difficulties with standard therapy development for rare diseases, an alternative experimental method is the personalized (N-of-1) approach, which involves creating a custom-designed for a single patient who has no alternative treatment options (80). The development of drugs targeting rare genetic diseases prompting extensive research efforts focused on discovering novel compounds and identifying new molecules (**Figure 3**).

Recently, antisense therapy has become as a promising treatment strategy for rare diseases, especially in the context of N-of-1 therapy development (81, 82). The sequence-specific ASOs binding provides exceptional precision in N-of-1 approaches, where the oligonucleotide can be targeted to a patient’s exact mutant sequence. ASOs are being studied worldwide as unique candidates for developing therapies for N-of-1 therapies (81).

Here we provide an overview of the antisense developed that currently available, and describe potentials of antisense-based therapeutics for the treatment of rare and «ultrarare» endocrine diseases.

### Molecular therapy as potential therapeutic strategy for medullary thyroid carcinoma

Medullary thyroid carcinoma is a rare neuroendocrine tumor that arises from parafollicular C cells of the thyroid and represents only 1% to 2% of all thyroid malignancies (83). It manifests either sporadically (in 75% of cases) or in a hereditary manner: multiple endocrine neoplasia type 2 (MEN 2), familial medullary thyroid carcinoma (84). While many patients may experience a relatively slow progression of the disease even in the presence of metastasis, the 10-year survival rate from time of first metastasis ranges from 10% to 40% (83). Due to its early dissemination and the absence of effective systemic therapy, medullary thyroid carcinoma has a poorer prognosis compared to follicular and papillary thyroid cancers (85).

The primary treatment for medullary thyroid carcinoma includes appropriate thyroidectomy and removal of affected cervical lymph nodes. Postoperative radiotherapy may improve local control of the disease (85). Moreover, none of the currently available systemic therapies provide cure, and these treatments often have significant potential side effects.

A key characteristic of many cancers is the suppression of apoptosis, which typically leads to genetic instability and activates an apoptotic response in healthy cells (86). Increased levels of various inhibitors of apoptosis protein family members have been identified in many cancers and over-expression of these proteins contributing to resistance against apoptotic signals in various malignancies (87). There is a concerted effort to investigate the specific role of inhibitors of apoptosis proteins in tumorigenesis and to explore their potential as therapeutic targets for cancer treatment (88, 89). Among these proteins, survivin has taken a center stage due to its highly specific expression in cancer cells (90). Survivin inhibits apoptosis both *in vitro* and *in vivo*, potentially through its interactions with multiple regulators of intrinsic and extrinsic apoptotic pathways. Its selective overexpression in a number of cancers, including lung, breast, colon, brain, gastric, esophageal, pancreatic, liver, uterine and ovarian cancer cells, has been strongly associated with tumor progression, therapeutic resistance, and poor patient prognosis (91). High levels of survivin expression have also been observed in thyroid cancer cell lines resistant to cisplatin (92). These unique properties make survivin as an attractive target for the development of innovative cancer therapies aimed at overcoming resistance to conventional treatments.

The first attempt at ASO therapy targeted at survivin successfully induced apoptosis in human melanoma cell lines (93). Recent studies confirmed that chemically synthesized oligonucleotides can specifically inhibit survivin at both mRNA and protein levels.

The expression of survivin was immunohistochemically determined in 10 paraffin-embedded tissue samples of human normal thyroid and in 10 samples of medullary thyroid carcinoma, as well as in human medullary thyroid carcinoma cell line TT (TT cells). A positive incidence of surviving protein immunoreactivity was observed in 80% (8 out of 10 samples) in medullary thyroid carcinoma cells and a high expression of suvivin in TT cells, whereas expression of this protein was not detected in normal thyroid tissue. The expression of survivin in TT cells was detected at mRNA level by reverse transcription and polymerase chain reaction (RT-PCR) and confirmed at protein level by Western blot analysis. In this study, ASOs significantly reduce survivin gene expression at mRNA and protein levels, beginning within 12 hours and persisting over 48 hours after the start of transfection concomitant, with a decrease in viability and growth of TT cells in a dose-dependent fashion (94). Therefore, survivin-targeted molecular therapy using ASOs in combination with other therapeutic strategies may be a promising option for medullary thyroid carcinoma treatment.

siRNA-based survivin inhibitors have shown considerable promise in preclinical cancer models. For example, Paduano et al. (95) demonstrated that silencing of survivin gene by siRNAs effectively reduce tumor cell proliferation and enhanced the rate of caspase-9-dependent apoptosis in human androgen-independent prostate cancer cells. These results support previous observations indicating that interference with survivin function by the use of siRNAs (96–99) and other kinds of inhibitors, including ASOs, led to increased apoptotic cell death in different human tumor models. Moreover, several *in vitro* and *in vivo* studies indicated that survivin down-regulation sensitize human tumor cells to chemotherapeutic agents such as cisplatin, doxorubicin, etoposide and paclitaxel (100–102). These findings highlight the synergistic potential of combining siRNA with conventional treatments. In another study by Kappler et al. (96), survivin-specific siRNA knockdown the expression of survivin by 73–88% and survivin protein expression by 52–81% in five human sarcoma cell lines regardless of the presence or absence of wild-type p53 alleles. This finding was coupled with a reduction in clonogenic survival ranging from 65–86%.

Survivin-targeting therapies using ASO and siRNA hold immense potential for advancing cancer treatment. While specific siRNAs targeting survivin have not been tested on medullary thyroid carcinoma, they may be repurposed for this disease since ASO-based survivin inhibitor has proved the concept. However, before using RNA interference technology in human clinical trials, significant efforts must be undertaken to ensure the specificity of siRNAs and to improve safe and effective delivery systems. In this regard, the potential to transform survivin inhibitors into drug-like small molecules may provide opportunities for their clinical use (103).

### New therapeutic agents for acromegaly

Acromegaly is a rare disease caused by increased growth hormone secretion due to a pituitary adenoma, resulting in increased levels of circulating insulin-like growth factor 1 (IGF-I). The disease is associated with systemic complications which deleteriously affect quality of life, increase morbidity and mortality (104). Advancements in the treatment of acromegaly involving new treatment options provide more effective multidisciplinary and patient-oriented approach. ASOs targeting the growth hormone receptor (GHR) may be a novel therapy for acromegaly.

**Figure 4:**
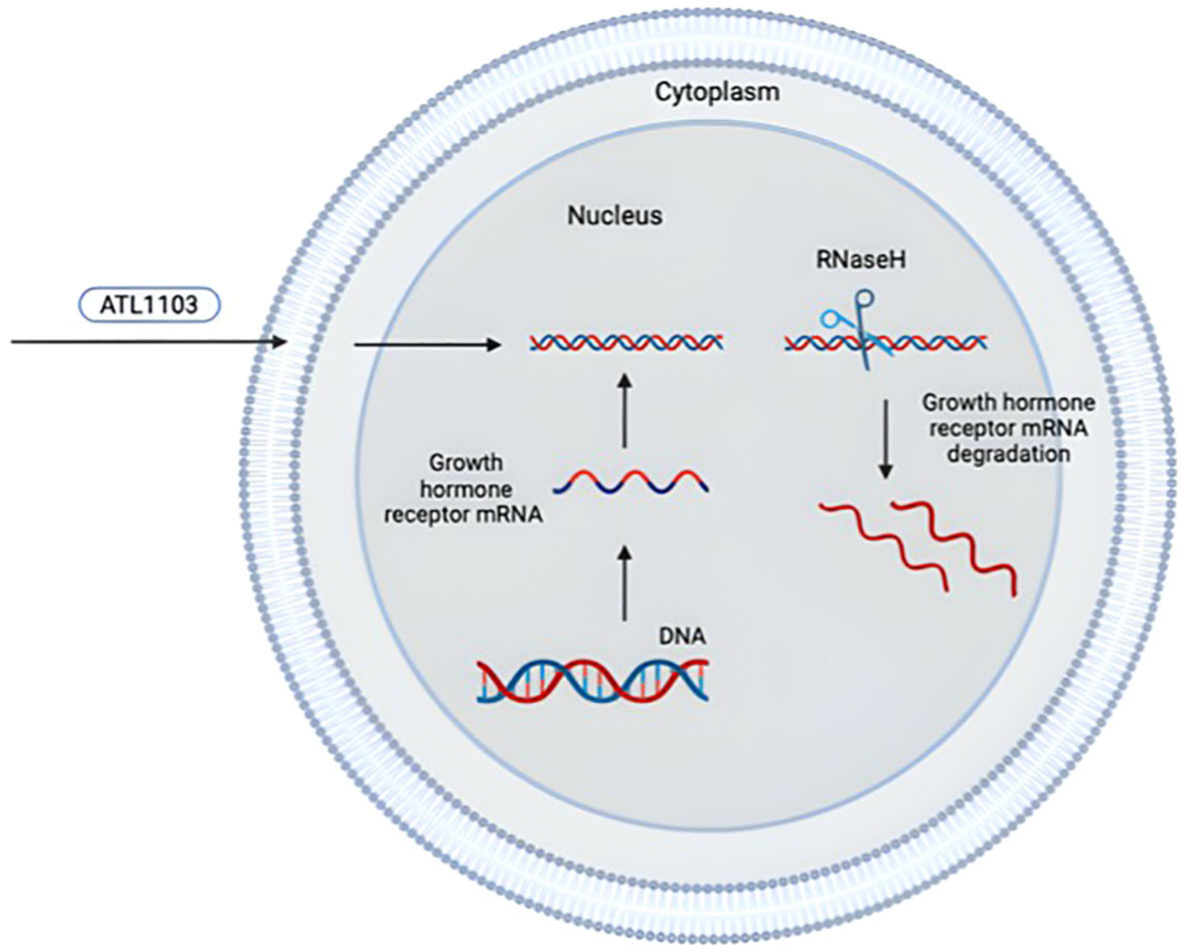
Mechanism of antisense inhibition of ATL1103 [adapted from (105)]. The drawing was created using BioRender web-tool.

ATL1103 is a second-generation ASO designed to inhibit translation of human GHR mRNA and developed by the Australian company Antisense Therapeutics. ATL1103 is administered by subcutaneous injection (once or twice a week). Antisense therapy is based on single-stranded synthetic oligonucleotides that combine with GHR mRNA and convert it into a substrate for RNaseH. As a consequence, the reduction of mRNA translation to protein leads to reduced GHR synthesis (**Figure 4**) (105).

In preclinical studies on rodents and primates, subcutaneous injections of ASO reduced GHR mRNA levels, growth hormone binding activity to the liver cells by 34% and serum IGF-I levels by 44% in mice after seven days of dosing compared with saline-treated mice (106). A randomized, placebo controlled, double blind study phase I in healthy 24 adult male demonstrated a trend in reduction IGF-I levels with a significant effect at day 21 with a 7% reduction in mean IGF-I levels versus baseline. Moreover, ATL1103 had a significant effect on reducing growth hormone–binding protein at day 21 (https://www.asx.com.au/asxpdf/20111207/pdf/4234016x2cj5xn.pdf).

In a randomized open-label parallel-group study phase II, evaluating the potential of ATL1103 as a treatment for acromegaly, 26 patients with active stage of the disease were randomized to subcutaneously administer ATL1103 at a dose of 200 mg once or twice a week for 13 weeks. This study showed that use of ATL1103 twice a week resulted in a median fall in serum IGF-I by 27.8% (range 4.4 – 49.8%, p = 0.0002) at week 14 compared to the baseline level, while no change was seen with once-weekly dosing. ATL1103 was well tolerated, with injection-site reactions being the most common treatment adverse events, observed in 85% of patients. There was no clinically significant increase in the pituitary tumor volume, however, the short duration of treatment and follow-up does not allow to draw definitive conclusions about the long-term effect of ATL1103 on this parameter (107).

Cimdelirsen (IONIS-GHR-LRx; ISIS 766720) – another novel, ligand-conjugated, hepatic-targeted investigative antisense molecule. Cimdelirsen targets GHR mRNA and reduces GHR synthesis and the amount of GHR on cell membrane. Cimdelirsen was evaluated in a 4-month double-blind, placebo-controlled phase 2 study in uncontrolled acromegaly patients (IGF-1 between 1.3 to 5 x upper limit of normal) treated with long-acting somatostatin receptor ligands (NCT03548415) and in an open-label extension safety study (NCT03967249). Once monthly subsequently Cimdelirsen injections demonstrated long-term safety, were well-tolerated, and resulted in significant reductions in growth hormone binding protein (-2% placebo, -43% low dose, -64% high dose; p<0.001) and IGF-1 AUC without increased growth hormone levels (108).

### Molecular therapies for isolated growth hormone deficiency type II

Isolated growth hormone deficiency is a rare congenital disorder with a ranging from 1:4000 to 1:10000 live births. Up to 30% of primary cases appear to be familial, while the majority are sporadic (109). Newborns with a genetic cause of isolated growth hormone deficiency have poor linear growth that gradually worsens with age, and typically share several clinical characteristics (110). Isolated growth hormone deficiency can be divided into four types based on the inheritance pattern and clinical features, including autosomal recessive (types IA and IB), autosomal dominant (type II), or X-linked (type III) (110). Key genes associated with the genetic basis of the disease include gene coding for growth hormone (GH-1) or the growth hormone-releasing hormone receptor (GHRHR). Additionally, isolated growth hormone deficiency can be caused by mutations in one of several transcription factors, such as *HESX1*, *OTX2*, *PROP1*, *POU1F1*, *SOX2* and *SOX3* (111).

Most mutations leading to isolated growth hormone deficiency type II affecting splicing of the GH-1 gene. When GH-1 is correctly spliced, it produces the 22kDa isoform, which is the primary biologically active circulating form of growth hormone. Nevertheless, even under normal conditions, a small percentage of alternative splicing products are generated, including 20kDa isoform, 17.5kDa isoform and severely truncated 11.3kDa and 7.4kDa isoforms (111). The mutations lead to the skipping of exon 3 during splicing, resulting in the production of a shorter variant of growth hormone variant known as the 17.5kDa of growth hormone isoform, which functions as a dominant negative isoform and inhibits secretion of the full-length 22kDa of growth hormone isoform (111, 112). The disease severity correlates with the levels of properly spliced RNA and the ratio of alternatively spliced isoforms (113). Moreover, when the 17.5kDa isoform is overproduced, it also leads to destruction of neighboring cells by invasion by macrophages, resulting to severe hypoplasia and deficiencies of additional anterior pituitary hormone (114).

Children diagnosed with isolated growth hormone deficiency type II receive daily injections of recombinant human growth hormone in order to reach normal height. However, such treatment cannot fully replicate the normal pulsatile pattern of growth hormone secretion and does not prevent toxic effects of 17.5kDa isoform on the pituitary gland, potentially leading to additional hormonal deficiencies (115). Consequently, therapies specifically targeting the harmful 17.5kDa growth hormone isoform may prove beneficial for patients with GH-1 splicing defects.

siRNAs can be designed to specifically target these aberrantly spliced isoform for degradation. In 2004, Ryther et al. (116) suggested using siRNAs that specifically target mRNA of the 17.5kDa isoform. They designed siRNA to target the junction between exon 2 and exon 4, as this sequence is unique to the transcripts encoding the 17.5kDa isoform. Their findings demonstrated that plasmids expressing this siRNA effectively degraded the exon 3-skipped growth hormone transcripts in cultured GH3 cells by greater than 90%.

Later, Shariat et al. (117) successfully used RNA interference (RNAi) to specifically target the mutant growth hormone-1 transcript encoding the 17.5kDa isoform *in vivo*, allowing recovery of wild-type growth hormone levels to rescue an autosomal dominant mouse model of human isolated growth hormone deficiency type II. Mice expressing short hairpin RNA (shRNA)-17.5 were bred with the isolated growth hormone deficiency type II mice. The resulting transgenic mice exhibited both macroscopically and microscopically normal somatotroph populations in the rescue mice, leading to full functional recovery without any overt phenotype in other cells. However, when mice are crossed, only the progeny that contain both the shRNA transgene and transgene encoding the 17.5kDa isoform (Δ3 transgene) exhibit the rescue phenotype. Moreover, it was shown that in the isolated growth hormone deficiency type II mice the dominant-negative hGH 17.5kDa protein is the predominant isoform, whereas in the rescue mice the major protein identified is the wild-type mouse growth hormone.

Above mentioned studies highlighted the effectiveness of siRNA or shRNA against the 17.5kDa isoform levels without affecting the normal 22kDa provides a promising approach to treat isolated growth hormone deficiency type II in humans (118). Nonetheless, effective exogenous delivery of siRNAs remains unachieved. A reliable and safe delivery system is needed for clinical application, since direct exposure to the pituitary gland is still relatively difficult due to its anatomical position.

### Possibilities of gene-based therapies for the treatment of osteogenesis imperfecta

Osteogenesis imperfecta includes a diverse group of inherited connective tissue disorders, characterized by bone fragility and a wide range of phenotypes. Advances in molecular biology and radiological methods during the late 1970s facilitated the establishment of a classification system for osteogenesis imperfecta based on clinical manifestations (119). The latest classification of genetic skeletal disorders, defined in 2019, distinguishes five types of osteogenesis imperfecta, including the first four types (types I–IV) from the original Sillence’s classification, and type V, which is characterized by calcification of interosseous membranes and/or hypertrophic callus (120, 121).

The majority of patients (85%) with osteogenesis imperfecta have an autosomal dominant mutation in genes responsible for the synthesis of type I collagen, specifically *COL1A1* and *COL1A2*. Two primary mutations in these genes are linked to either structural or quantitative defects in the production or processing of type I collagen. In addition, variants of the disease with autosomal recessive and X-linked inheritance have also been identified, involving other genes (122–124).

Current treatment options for osteogenesis imperfecta focus on fracture prevention, symptom management and increasing bone mass. The progression of novel treatment modalities and therapeutic approaches for osteogenesis imperfecta primarily depends on a better understanding of molecular mechanisms and genetic background of the disease (125).

Correction of genetic defects may be potential treatment for osteogenesis imperfecta. There are several approaches for modifying collagen mutant transcripts. One novel strategy aims to transform the severe form of osteogenesis imperfecta, characterized by structural defects in collagen type I, to the mild type caused by a quantitative defect in normal collagen through the allele-specific gene silencing. Multiple approaches can be employed to silence collagen mutant transcripts. For example, the use of ASOs and siRNAs have been tested in various studies involving *in vitro*, *ex vivo* and, to a lesser degree, animal models of osteogenesis imperfecta.

The principle of allele specific silencing of collagen type I genes was explored in 1996 by Wang Q. and Marini JC (126). who used cultured fibroblasts from a patient with type IV osteogenesis imperfecta. Significant suppression of the mutant protein chain and mRNA was achieved with ASO to both mRNA and nuclear levels. Mutant protein was suppressed to 44–47% and mutant alpha 2(I) mRNA to 37–43% of their levels in control cells, but the suppression achieved was insufficient for clinical intervention.

Millington-Ward et al. (127) evaluated RNA interference (RNAi) as a means to downregulate *COL1A1* expression in Cos-7 cells and in human mesenchymal progenitor stem cells. Preferential suppression of individual polymorphic alleles that differed by a single nucleotide was observed.

In 2008 Lindahl et al. (128) published a study in which they examined the allele dependent effects of seven tiled siRNAs targeting a region surrounding an exonic *COL1A2* T/C polymorphism (rs1800222) in heterozygous primary human bone cells. In a study published later by Lindahl et al. (129) it was attempted to investigate an approach based on the allele-preferential silencing of collagen type I. The allele discriminatory effects of siRNAs targeting each allele of 3’UTR insertion/deletion polymorphisms (indels) in *COL1A1* (rs3840870) and *COL1A2* (rs3917) have been studied in primary human bone derived cells obtained from individuals with heterozygote genotypes for the indels. In cells treated with siRNAs targeting the *COL1A1* alleles mRNA levels were reduced by 65% and 78% compared to negative control levels, and in cells treated with *COL1A2* siRNAs mRNA levels were reduced by 26% and 49% compared to corresponding negative controls (129).

Rousseau et al. (130) demonstrated *in vitro* and *ex vivo* that an RNAi approach to allele-specific gene silencing using Mut exonic sequence and the Mut primary fibroblasts from the osteogenesis imperfecta Brtl murine model with si/shRNAs can be designed and successfully applied *in vitro* to allele-specific suppression of type I collagen gene.

While these findings are promising, it is important to note that these techniques remain in the experimental phase. Moreover, no *in vivo* targeting of bone was attempted, which remains a notable limitation. Several issues still need to be addressed, including the specific design of silencing molecules and the development of carrier agents for their delivery to bone. Due to the different locations of mutations responsible for osteogenesis imperfecta, the creation of universal silencing molecules is very difficult. Additionally, the duration of the therapeutic effects remains unknown that require clinical trials.

### Potential molecular therapy for lipodystrophy syndromes

Lipodystrophy syndromes represent a heterogeneous group of disorders characterized by a deficiency of adipose tissue affecting the whole body (generalized lipodystrophy) or specific regions (partial lipodystrophy) depending on the type of lipodystrophy (131). The estimated global prevalence of lipodystrophy syndromes is 1,3–4,7 cases per million (132). The classification of lipodystrophies is based on physical characteristics, distinguishing between partial and general forms, and distinguishing inherited from acquired variants. There are four major categories: congenital generalized lipodystrophy, familial partial lipodystrophy, acquired generalized lipodystrophy, and acquired partial lipodystrophy (133).

Congenital generalized lipodystrophy syndromes are autosomal recessive diseases, mainly due to null variants in *AGPAT2*, involved in the glycerophospholipid/triacylglycerol biosynthesis pathway (134), or in *BSCL2* which encodes a 398-amino acid integral endoplasmic reticulum membrane protein called seipin that take part in lipid droplet formation and adipocyte differentiation (135). Congenital generalized lipodystrophy type 3 and congenital generalized lipodystrophy type 4 are due to genetic mutations in caveolin-1 and cavin-1, respectively, both of which are crucial for the formation of caveolae, specialized microdomains of the cell plasma membrane that activate various signaling pathways. Additionally, caveolin-1 and cavin-1 are found in adipocyte lipid droplets, facilitating intracellular lipid transport (136). Most familial partial lipodystrophies are inherited as autosomal dominant disorders, often resulting from loss-of-function or dominant negative mutations. While familial partial lipodystrophy type 1 is likely a multigenic form of lipodystrophy syndrome, familial partial lipodystrophy type 2, caused by pathogenic variants in the *LMNA* gene, represents the most common genetically determined form of partial lipodystrophy (137). Generalized or partial lipoatrophy resulting from genetic mutations in DNA repair manifest clinically as progeroid syndromes, characterized by signs of premature aging, lipodystrophy, insulin resistance, and metabolic disturbances.

Lipodystrophy syndromes are heterogeneous and are diagnosed based on a clinical phenotype, supplemented by genetic testing in some cases. Genotyping may include sequencing of a limited number of candidate genes, panel of candidate genes, or whole-exome/whole-genome sequencing. The pathophysiology of lipodystrophy involves dysfunctional adipocyte differentiation, impaired triglyceride integration, and errors in transcription and translation processes governing adipogenesis (133).

Lipodystrophy syndromes are usually manifested by several complex metabolic complications such as insulin resistance and diabetes mellitus, hypertriglyceridemia, non-alcoholic fatty liver disease, which can lead to organ damage (133, 138). Current therapies aim to improve or prevent long-term metabolic complications and organ damage. There is no cure for lipodystrophy and no therapy that could restore the growth of adipose tissue. The standard clinical approach is based on lifestyle modification and the use of hypoglycemic, lipid-lowering and cardiovascular drugs to treat specific concomitant metabolic diseases (133).

Therapeutic approaches for treating lipodystrophy have been primarily explored in clinical trials targeting familial partial lipodystrophies. The most common treatment approach is metreleptin therapy, which has been extensively researched and used as a replacement for leptin (131). Metreleptin (r-metHuLeptin), administered by subcutaneous injection once daily, is an analog of human leptin made through recombinant DNA technology. Treatment with metreleptin is well tolerated and resulted in improvements in glycemic control, hypertriglyceridemia, and liver volume (139). Although metreleptin does not lead to the restoration of lacking adipose tissue, it has been proven to be effective in generalized lipodystrophy syndromes.

Due to the rarity of the disease, understanding of this condition is limited, and therefore effective therapeutic treatment options for lipodystrophy syndromes remain very limited.

The potential therapeutic targets for ASO and siRNA include genes involved in adipocyte differentiation, lipid metabolism, insulin sensitivity and inflammatory processes, as well as those linked to specific mutations in lipodystrophy-associated genes. These molecular therapies could help restore normal fat distribution, improve lipid metabolism and correct insulin resistance, which are central features of the disease. One of the primary areas for therapeutic intervention is genes involved in adipocyte differentiation. *PPARG* (peroxisome proliferator-activated receptor gamma) is a key regulator of adipogenesis. Loss-of-function pathogenic variants in *PPARG* impair the differentiation of preadipocytes into adipocytes, leading to fat loss and severe metabolic consequences associated with familial partial lipodystrophy type 3 (140). Targeting *PPARG* with ASO could help restore normal adipocyte function and fat storage. Similarly, *LMNA* (lamin A/C), encoding a protein essential for nuclear structure, is frequently mutated in lipodystrophy, leading to adipocyte degeneration. siRNA targeting to suppress the expression of mutant forms of *LMNA* could stabilize adipocyte nuclei and prevent cell loss. Another important gene is *AKT2* (protein kinase B, alpha), which is involved in insulin signaling and adipocyte differentiation. siRNA designed to restore *AKT2* function could enhance both adipogenesis and insulin sensitivity, which is crucial for managing the metabolic aspects of lipodystrophy (141).

Lipid metabolism dysregulation is another hallmark of lipodystrophy syndromes. Leptin deficiency with resultant hyperphagia coupled with inadequate fat storage results in metabolic complications of lipodystrophy. Apolipoprotein C-III (apoC3) and angiopoietin-like protein 8 (ANGPTL8), recognized as inhibitors of lipoprotein lipase, play a role in modulating hypertriglyceridemia. The BROADEN study (A Study of Volanesorsen (Formerly IONIS-APOCIII_Rx_) in Participants With Familial Partial Lipodystrophy) which was the largest global double blinded placebo controlled study published in 2022 demonstrated that treatment with volanesorsen (ISIS 304801/ISIS-APOCIII_Rx_/IONIS-APOCIII_Rx_, Waylivra^®^, Akcea/Ionis), an antisense oligonucleotide to apo-CIII, reduced serum triglyceride levels by more than 88% from baseline at 3 months and improved hepatic steatosis by 53% at month 12 with volanesorsen versus placebo (142).

The main APOC3 inhibitors in advanced clinical development are the GalNAc-conjugated ASO targeted to hepatic APOC3 mRNA to inhibit apoC3 production (Olezarsen (ISIS 678354, IONIS-APOCIII-L_Rx_, AKCEA-APOCIII-L_Rx_, Akcea/Ionis)) and the GalNAc-siRNA Plozasiran (APOC3-targeted siRNA drug for lowering triglyceride and APOC3) (143, 144). Moreover, genes that regulate lipid metabolism also represent important targets for ASO and siRNA therapies.

In lipodystrophy, insulin resistance is a major metabolic issue, especially in generalized forms of the disease. Key genes involved in insulin signaling pathways, such as *INSR* (insulin receptor) and *IRS1* (insulin receptor substrate 1), could serve as targets for therapy. siRNA targeting *INSR* could potentially restore receptor function and improve insulin sensitivity. Similarly, *IRS1*, which transmits signals from the insulin receptor, is another promising target. ASOs modulating *IRS1* expression may be possible to restore proper insulin signaling and reduce insulin resistance, improving the metabolic state of the patient (145).

The use of ASOs and siRNA represents a promising therapeutic approach for people suffering from lipodystrophy. However, the clinical translation of these technologies requires overcoming challenges such as efficient tissue-specific delivery, minimizing off-target effects and ensuring long-term efficacy. Further studies are required to develop molecular therapy in order to provide effective treatment for lipodystrophy syndromes and related metabolic disorders, offering targeted and personalized solutions.

## Conclusion

Molecular therapy seems to be a very promising strategy for the future treatment, especially for endocrine diseases. ASOs drugs and RNA-based drugs have unique capabilities for use in target groups of patients or can be tailored as patient-customized N-of-1 therapeutic approach. Furthermore, there is also a hope that new drugs based on ASOs or RNA-interference will be developed to target proteins and pathways altered in some forms of monogenic diabetes, monogenic and syndromic obesities. This demonstrates the significant potential of these therapeutic strategies, which promise to be effective in a wide range of currently untreatable disorders. However, the expected clinical benefits should always outweigh the therapeutic risks. As our understanding of disease pathogenesis expands, antisense therapy now can be used not only for the treatment of monogenic diseases, but also for the treatment of polygenic diseases. We hope that more and more patients with endocrine diseases will be able to receive molecular therapy soon.
